# Vaccination with a Live Avirulent *E. coli* Vaccine Resulted in Improved Production Performance Combined with a Significant Reduction in Antimicrobial Use

**DOI:** 10.3390/antibiotics14060547

**Published:** 2025-05-27

**Authors:** Frédéric Vangroenweghe, Thomas Matthijs, Marnix Sinnaeve

**Affiliations:** 1 Elanco Animal Health Benelux, BU Swine & Ruminants, Generaal Lemanstraat 55/3, 2018 Antwerpen, Belgium; 2 Unit of Porcine Health Management, Department of Internal Medicine-Reproduction-Population Medicine, Faculty of Veterinary Medicine, Ghent University, Salisburylaan 133, 9820 Merelbeke, Belgium; 3 DAP Vartos, Mandellaan 373, 8800 Roeselare, Belgium; thomas.matthijs@ostynvoeders.be (T.M.); marnix.sinnaeve@ostynvoeders.be (M.S.)

**Keywords:** post-weaning diarrhea, antimicrobial coaching trajectory, live oral *E. coli* vaccination, antimicrobial reduction

## Abstract

Background/Objectives: In swine production, the post-weaning period has been identified as one of the most challenging and stressful periods in the life of a piglet due to changes in its environment and feeding regimen. During this period, piglets might undergo infectious challenges with enterotoxigenic *Escherichia coli* (ETEC) resulting in post-weaning diarrhea (PWD), and meningitis due to *Streptococcus suis*. Therefore, metaphylactic and curative antimicrobial therapy is frequently applied, which leads to an increased treatment incidence per 100 days at risk (TI_100_). Methods: Here, we report the results of an antimicrobial coaching trajectory in a 1000-sow farm with high antimicrobial use during the post-weaning period. For a period of 21 weeks, we evaluated the effect of an oral live avirulent *E. coli* F4F18 vaccine (Coliprotec^®^ F4F18; Elanco AH) for the active immunization of piglets against PWD caused by F4- and F18-ETEC on the reduction in antimicrobial use during the post-weaning period. A 1000-sow farm with PIC sows operating in a 1-week BMS was rated as an ‘attention farm’ at the level of the post-weaning period according to the Antimicrobial Consumption and Resistance in Animals (AMCRA) benchmark reporting tool. To analyze the specific approach towards antimicrobial use and the related post-weaning pathology, a farm visit including a biosecurity check was carried out together with all associated stakeholders. Subsequently, an antimicrobial coaching trajectory was utilized to follow-up on the improvement of the reduction in antimicrobial use after implementation of the various pieces of advice. Results: For analytical purposes, we compared the results obtained in period 1 (P1; vaccination week 1–6) to period 2 (P2; vaccination week 7–21), since practical field experience has demonstrated that a ‘stabilization period’ of about 6 weeks is necessary to obtain the maximal effect of vaccination. There was a significant reduction in mortality (5.7% to 2.0%) and improvement in the average daily weight gain (366 g/d to 392 g/d) following vaccination, with a simultaneous reduction in the number of days in nursery (45 days to 38 days). Meanwhile, the weight at the end of nursery remained at a similar level. There was a clinically relevant though non-significant decrease in the TI_100_ (32.8 days to 20.6 days). Overall, the implementation of all measures resulted in a positive ROI of 2.72 per piglet. Conclusions: The implementation of several biosecurity measures in combination with the use of an oral live avirulent *E. coli* F4F18 vaccine (Coliprotec F4F18) could improve performance parameters and reduce mortality, while reducing the number of days in nursery and the TI_100_. Overall, a positive return on investment of 2.72 could be obtained per piglet produced under these improved conditions.

## 1. Introduction

In swine production, the post-weaning period has been identified as one of the most challenging and stressful moments in the life of a piglet due to changes in its environment and feeding regimen. During this phase, piglets might undergo infectious challenges with enterotoxigenic *Escherichia coli* (ETEC) resulting in post-weaning diarrhea (PWD) [1], and *Streptococcus* (*S.) suis* leading to polyserositis, including arthritis, peritonitis, pleuritis, pericarditis, and meningitis [2]. Therefore, metaphylactic and curative antimicrobial therapy is frequently applied, which leads to an increased treatment incidence per 100 days at risk (TI_100_) [3]. The calculation of the TI_100_ is based several parameters including the total amount of antimicrobial used, the animal daily dose of the concerned products, the total weight of animals at risk, the number of days at risk, and the duration of activity of the antimicrobial (long-acting factor).

Post-weaning diarrhea (PWD) in pigs remains an economically important disease [1] and it is characterized by reduced piglet performance (i.e., mortality, weight loss, retarded growth, batch-to-batch variation) and increased treatment costs (i.e., higher antimicrobial use) [4,5,6,7,8,9,10]. The enterotoxigenic *E. coli* (ETEC) pathotype—characterized by the presence of fimbrial adhesins that mediate attachment to porcine intestinal enterocytes, and enterotoxins which disrupt fluid homeostasis in the small intestine—is regarded as the most important cause of PWD. These interactions with the intestinal enterocytes result in mild-to-severe diarrhea within a few days post-weaning. This is associated with clinical signs of dehydration, the loss of body condition, and mortality [11]. The most commonly occurring virulence factors responsible for the PWD pathology due to ETEC in pigs are the adhesive fimbriae F4 (K88) and F18 [11]. Other fimbriae such as F5 (K99), F6 (987P), and F41 rarely occur in *E. coli* isolates from PWD [11,12,13,14,15]. The other virulence factors are the enterotoxins associated with porcine ETEC, namely heat-labile toxin (LT), heat-stable toxin a (STa), and heat-stable toxin b (STb). Some pathogenic strains might produce both enterotoxins and a Shiga toxin (Stx2e) [11].

The disease is currently controlled using antimicrobials, such as colistin and lincomycin–spectinomycin, although the emergence of antimicrobial resistance in *E. coli* strains isolated from cases of PWD urges the need for alternative control measures [16,17,18,19,20].

Several alternative nutritional strategies such as supplementary dietary fiber, a reduction in the crude protein level, improved ingredient consistency, the addition of pre- and probiotics, and acid supplementation have been explored to increase intestinal health and decrease the incidence of PWD due to *E. coli* in post-weaned piglets [21,22,23,24,25,26,27,28,29]. Depending on the infection pressure in the herd, these strategies had a variable effect on both intestinal health and the disease incidence of PWD.

Other preventive strategies have recently been explored [1,30]. For an *E. coli* vaccination against PWD due to F4- and F18-ETEC, it is important to activate mucosal immunity against F4 and F18 through the local production of F4- and/or F18-specific secretory immunoglobulin A (sIgA) antibodies. These local antibodies can prevent pathogenic F4- and F18-ETEC from attaching to the intestinal F4 and F18 receptors and thus reduce the clinical signs of PWD [30]. Recently, vaccination with an oral live avirulent *E. coli* F4 or *E. coli* F4 and F18 vaccine has demonstrated efficacy against PWD due to F4-ETEC and F4- and F18-ETEC [31,32]. This immunization resulted in the decreased severity and duration of PWD clinical signs and fecal shedding of F4- and F18-ETEC [31,32], and increased weight gain in piglets vaccinated with an *E. coli* F4 vaccine [31]. Therefore, these positive effects of vaccination to protect post-weaned piglets against PWD due to *E. coli* can help pig producers to meet the recently updated criteria on the prudent use of antimicrobials that were published (EY 2019/6) [33], without compromising piglet performance post-weaning [2,34].

The economic impact of PWD due to *E. coli* was reported to be as high as EUR40 per sow per year based on a prolonged rearing time (+3.5 days) and increased mortality (+1.5%) [35]. A more detailed calculation was published in 2021 [34] considering performance parameters (average daily weight gain (ADWG), feed conversion rate (FCR), and mortality) and the costs of antimicrobial treatment (TI_100_). Vaccinated piglets had an improvement in ADWG (+11 g/d), FCR (−0.06), and mortality (−3.10%) with a reduction in the TI_100_ (−15.06).

Here, we report the results of an antimicrobial coaching trajectory in a 1000-sow farm with high antimicrobial use during the post-weaning period. For a period of 21 weeks, we evaluated the effect of an oral live non-pathogenic *E. coli* F4F18 vaccine (Coliprotec^®^ F4F18; Elanco; Indianapolis, IN, USA) for the active immunization of piglets against PWD caused by F4- and F18-ETEC on the potential reduction in antimicrobial use and the economic impact on production results during the post-weaning period.

## 2. Results

### 2.1. Piglet Performance

Piglet performance results compared between P1 (1–6 wks; period of the start of vaccination) and P2 (7–22 wks; period of ongoing vaccination) are given in Table 1.

**Figure 1:**
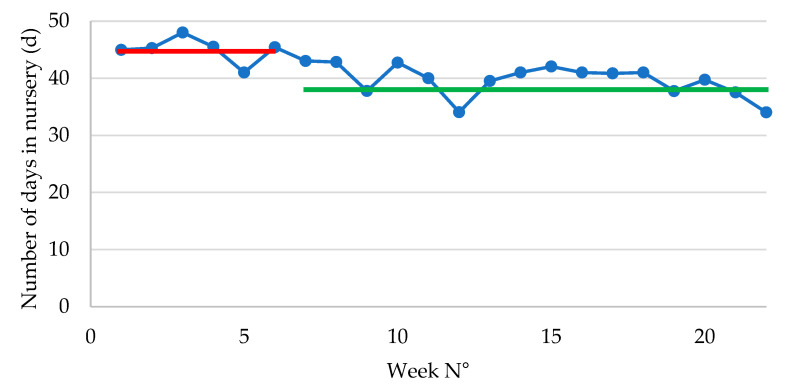
Number of days in the post-weaning phase in piglets following vaccination with an oral live avirulent *Escherichia coli* F4F18 vaccine (Coliprotec F4F18; Elanco Animal Health, Indianapolis, IN, USA) at 6 days pre-weaning. Red line indicates the average number of days in nursery (44.95 d) during P1 (1–6 weeks) of the *E. coli* vaccination. The green line indicates the average number of days in nursery (38.01 d) during P2 (7–22 weeks) of the *E. coli* vaccination.

The average weaning weight improved significantly (*p* = 0.001) following ongoing *E. coli* vaccination (6.40 ± 0.17 kg in P1 vs. 7.03 ± 0.07 kg in P2). The number of weaned piglets was numerically higher in P1 (n = 851 ± 79) as compared to P2 (n = 752 ± 29). The average number of days in nursery was significantly (*p* = 0.002) higher in P1 (44.95 ± 1.13 days) as compared to P2 (38.01 ± 0.74 days) (Figure 1).

**Figure 2:**
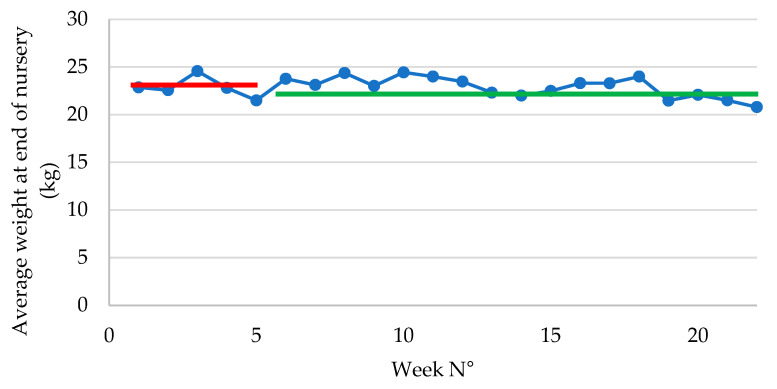
Average weight at end of post-weaning phase in piglets following vaccination with an oral live avirulent *Escherichia coli* F4F18 vaccine (Coliprotec F4F18; Elanco Animal Health, Indianapolis, IN, USA) at 6 days pre-weaning. Red line indicates the average weight at the end of nursery (22.86 kg) during P1 (1–6 weeks) of the *E. coli* vaccination. The green line indicates the average weight at the end of nursery (21.97 kg) during P2 (7–22 weeks) of the *E. coli* vaccination.

The average weight at the end of nursery was similar (22.86 ± 0.49 kg in P1 vs. 21.97 ± 0.26 kg in P2; *p* = 0.467) between both groups (Figure 2).

**Figure 3:**
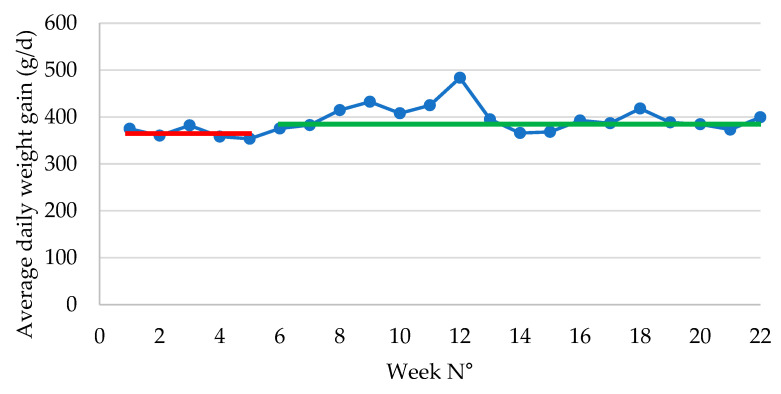
Average daily weight gain (expressed as g/d) in piglets during the post-weaning period following vaccination with an oral live avirulent *Escherichia coli* F4F18 vaccine (Coliprotec F4F18; Elanco Animal Health, Indianapolis, IN, USA) at 6 days pre-weaning. Red line indicates the average daily weight gain (362 g/d) during P1 (1–6 weeks) of the *E. coli* vaccination. The green line indicates the average daily weight gain (392 g/d) during P2 (7–22 weeks) of the *E. coli* vaccination.

The average daily weight gain significantly (*p* = 0.011) improved following ongoing vaccination (366 ± 5 g/d in P1 vs. 392 ± 7 g/d in P2) (Figure 3).

**Figure 4:**
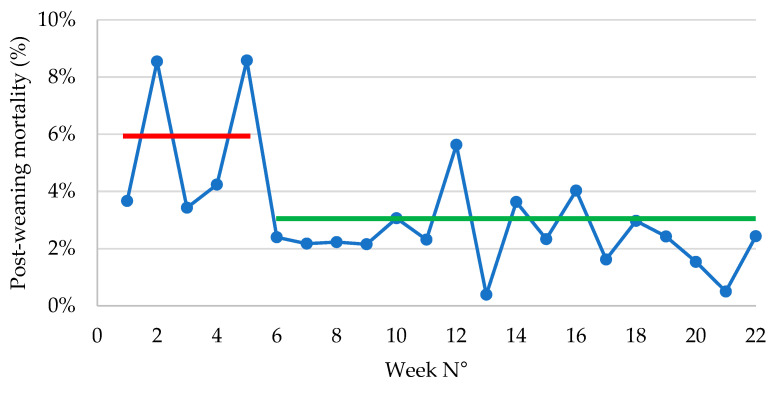
Percentage post-weaning mortality (expressed as %) of piglets during the post-weaning period following vaccination with an oral live avirulent *Escherichia coli* F4F18 vaccine (Coliprotec F4F18; Elanco Animal Health, Indianapolis, IN, USA) at 6 days pre-weaning. Red line indicates the average mortality percentage (5.7%) during P1 (1–6 weeks) of the *E. coli* vaccination. The green line indicates the average mortality percentage (2.0%) during P2 (7–22 weeks) of the *E. coli* vaccination.

Post-weaning mortality (expressed as the average number of dead piglets per weekly batch) during P1 (52 ± 11; 5.7 ± 0.01%) was significantly (*p* = 0.0001) higher as compared to P2 (16 ± 3; 2.0 ± 0.00) (Figure 4).

### 2.2. Antimicrobial Use—TI_100_

**Figure 5:**
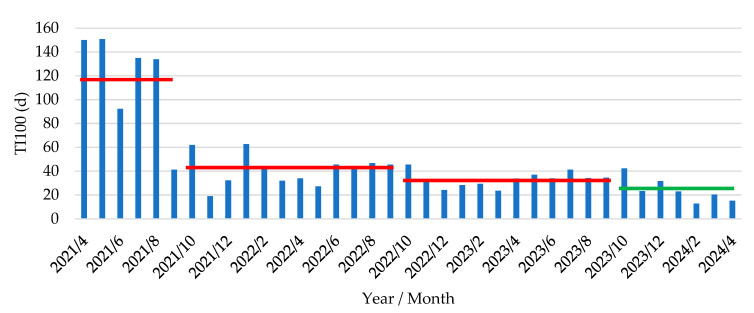
Treatment incidence over 100 days in post-weaning phase (TI_100_, expressed as d) in piglets following vaccination with an oral live avirulent *Escherichia coli* F4F18 vaccine (Coliprotec F4F18; Elanco Animal Health, Indianapolis, IN, USA) at 6 days pre-weaning. Vaccinated piglets were housed in the post-weaning facilities from October 2023 onwards. Red lines indicate the average TI_100_ over the last 30 months prior to the start of the *E. coli* vaccination (2021/4–2021/9, 117.2 ± 17.5 d; 2021/10–2022/9, 41.0 ± 3.8 d; 2022/10–2023/9, 33.2 ± 1.8 d). The green line indicates the average TI_100_ (24.1 ± 3.8 d) from the time point of *E. coli* vaccination onwards.

The antimicrobial molecules predominantly used during the post-weaning period consisted of colistin and the combination lincomycin–spectinomycin to treat PWD due to *E. coli* and amoxycillin to treat meningitis due to *S. suis.* The antimicrobial use, expressed as the TI_100_, was numerically but not significantly (*p* = 0.082) lower in P2 (20.6 ± 3.0 days) as compared to P1 (32.8 ± 9.6 days). Over a longer period, analysis of the TI_100_ was performed from the 2.5 years prior to the start of the *E. coli* vaccination to the period where piglets were vaccinated with the *E. coli* vaccine (including both P1 and P2). This comparison resulted in a significant (*p* = 0.028) difference between the period prior to *E. coli* vaccination (53.13 ± 7.00 days) and the period with *E. coli* vaccination (24.07 ± 3.83 days). The evolution of the TI_100_ from Q2 2021 until Q2 2024 is given in Figure 5.

**Figure 6:**
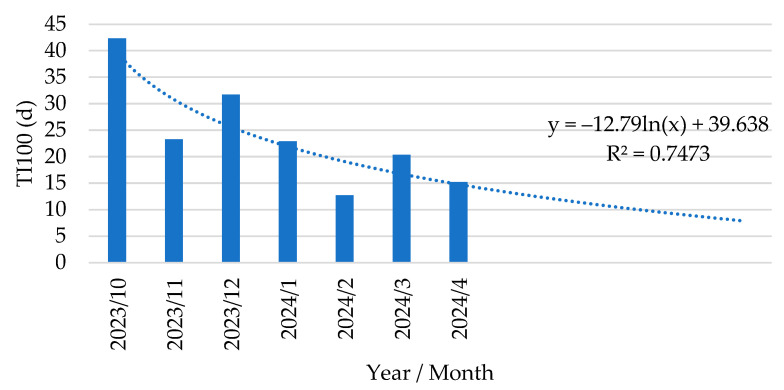
Trendline analysis of treatment incidence over 100 days in post-weaning phase (TI_100_, expressed as d) in piglets following vaccination with an oral live avirulent *Escherichia coli* F4F18 vaccine (Coliprotec F4F18; Elanco Animal Health, Indianapolis, IN, USA) at 6 days pre-weaning.

Based on the obtained reduction during the period with *E. coli* vaccination, a descending trendline was calculated with a R^2^ of 0.7473 (Figure 6).

### 2.3. Economic Return-on-Investment Results

A return-on-investment calculation was performed based on the model by [32] (Table 2). Performance data from P1 were labelled as the control data, whereas data from P2 were labelled as vaccine data. Following the ROI calculation, an extra gain per piglet following *E. coli* vaccination of +EUR2.72 was generated. For the specific 1000-sow farm with an average of 33 weaned piglets per sow per year, an extra income of +EUR89,746.80 was generated on a yearly basis.

## 3. Discussion

As soon as piglets are weaned, they need to develop active intestinal immunity due to the lack of passive lactogenic immunity. Therefore, vaccines should induce this protective effect by activating the mucosal immune system and production of antigen-specific immunoglobulins (IgA and IgM) [36]. Currently, three main types of vaccine strategy have been applied to protect post-weaned piglets against PWD due to ETEC. The first strategy is keeping the intestinal bacteria levels below the pathogenic level by intramuscular injectable vaccination. The second strategy is to block the adherence of ETEC through the oral administration of live attenuated or live wild-type non-enterotoxigenic *E. coli* strains with fimbrial adhesins that stimulate intestinal colonization by this *E. coli* strain, resulting in the induction of intestinal secretory antibodies. The third strategy is the reduction in fecal excretion of *E. coli* through the oral administration of purified fimbria, inducing a specific intestinal mucosal immune response [36]. Although there are many *E. coli* vaccines on the market to control swine enteric colibacillosis, only a few are registered for the prevention of PWD due to ETEC in post-weaned piglets. In Belgium, the only *E. coli* vaccine available on the market is a live avirulent *E. coli* F4 and F18 (Coliprotec F4F18; Elanco AH) vaccine that can be administered orally from 18 days of age.

The current study on the vaccination of pre-weaned piglets to protect against PWD due to F4-ETEC clearly demonstrates that the overall technical performance was significantly improved following continued vaccination with an oral live avirulent *E. coli* vaccine for several months, combined with a marked reduction in antimicrobial use throughout the vaccination period. Several economically important and clinically relevant performance parameters, such as the ADWG, number of days in nursery, and TI_100_, were significantly improved between the first 6 weeks of vaccination (P1; 1–6 weeks) and the subsequent 16 weeks of vaccination (P2; 7–22 weeks).

The total weight gain in nursery was approximately 890 g lower in P2 as compared to P1. Concurrently, the number of days in nursery reduced significantly from nearly 45 days in P1 to 38 days in P2, which implies that the piglets in P2 had a 7-day-shorter stay within the post-weaning facilities. Therefore, the ADWG was significantly improved at 26 g/d from 266 g/d in P1 to 392 g/d in P2. If the piglets remained the same number of days in nursery in both groups, the piglets in P2 would have an end-of-nursery weight of 24.67 kg, which would be much higher as compared to the end-of-nursery weight of 22.86 kg observed in P1.

During P2, a lower number of piglets were weaned per weekly batch as compared to P1. Considering the similar number of productive sows in each consecutive weekly batch, this would indicate a lower number of piglets weaned per sow in P2 as compared to P1. This might explain the significantly higher weaning weight (+630 g per piglet) registered during P2 (7.03 kg) as compared to P1 (6.40 kg).

From the perspective of antimicrobial use, there was a trend for a reduction in the TI_100_ between P1 and P2. There are several reasons for this gradual but ongoing decrease in the TI_100_ over time. Following a farm visit, several general and specific biosecurity measures were proposed with an impact on antimicrobial use during the post-weaning period. Water management resulted in a better water quality at the source level and improved waterline hygiene throughout the entire farm. Critical evaluation of the general hygiene protocols resulted in an improved cleaning and disinfection (C&D) protocol, following quantitative evaluation of the C&D results by Rodac plates. This improved C&D approach resulted in a reduction in the infection pressure throughout the farm facilities and, more specifically, within the post-weaning facilities. Besides the C&D protocol, needle management was upgraded, which resulted in less potential iatrogenic spread of disease pathogens through contaminated needles during treatment and vaccination procedures. The PWD-specific approach probably had the largest impact on the TI_100_ reduction, since adaptations in antimicrobial group treatment protocols were implemented in parallel to the vaccination. Standard antimicrobial treatment post-weaning was almost totally abolished both through the waterline and through feed premixes. When the clinical need for treatment was present at the pen level, the piglets in the specific pen were treated with a limited amount of antimicrobials using a water bowl instead of the previous protocol with an immediate intervention through an overall group treatment.

When analyzing the TI_100_ evolution over a longer period of 36 months prior to the start of the vaccination, an important reduction from 117.2 days (2021) to 33.2 days (2023) had already been obtained. However, under the current new AMCRA antimicrobial rules, the TI_100_ during the post-weaning period should not exceed 30 from 1 January 2025 onwards. Therefore, we can conclude that with all efforts made during the previous period, the farm that was originally categorized as an ‘attention farm’ at the end of 2023 was able to reduce its antimicrobial use to an acceptable level according to the AMCRA regulations.

In the current study, we compared piglets vaccinated with Coliprotec F4F18 during two consecutive periods, namely the first 6 weeks (P1) and the subsequent 16 weeks (P2), mainly due to the lack of reliable historical data on post-weaning performance in this farm. However, as we have experienced in the past, vaccination with Coliprotec F4F18 requires a few weeks (approx. 6 weeks) to stabilize the on-farm performance result. It was therefore logical to compare the results from these ‘artificially’ defined periods to observe the improvements in pig performance and antimicrobial use. If we had obtained reliable historical data, the difference between non-vaccinated piglets and vaccinated piglets would probably have been more pronounced. Another option could have been to run a concurrent control and treatment/vaccination group. However, due to several practical constraints related to housing during the post-weaning period, it would have been quite difficult to run this kind of trial set-up under the current field conditions. Moreover, piglets should be vaccinated prior to weaning, due to the 7-day onset of immunity to the oral live avirulent *E. coli* vaccine, and piglets of different litters were commingled according to their weight, quality, and general condition at weaning. Changes in these day-to-day routine management practices would complicate the set-up and performance of this practical field experience and might lead to involuntary and non-detectable errors that might have blurred the results and conclusions. Using the current study design allowed us to only change one specific parameter—i.e., the vaccination of piglets prior to weaning—and evaluate the effect of this implemented vaccination strategy over time in relation to the overall adaptations in management practices based on the antimicrobial reduction coaching trajectory

In Belgium, data on antimicrobial use at the farm and animal category level (sows, piglets, fattening pigs) are collected in a central database (Ab register, www.abregister.be, accessed on 27 April 2025) by the farm veterinarian on a quarterly basis. Based on these registrations and the individual delivery documents of the antimicrobial products, we could analyze the TI_100_ and details of all administered products (oral products both through drinking water and feed premix, and injectables) for PWD at the farm. The current study demonstrated a drastic decrease in antimicrobial use following the implementation of an oral live avirulent *E. coli* vaccine in piglets to prevent the clinical signs of PWD due to F4-ETEC. Indeed, both the overall TI_100_ and more detailed data per batch showed a marked decrease in antimicrobial use following vaccination. The average TI_100_ in the 2 years prior to implementation of the *E. coli* vaccine was 41.0 days (2022) and 33.2 days (2023), and this further decreased to 24.1 days upon implementation of *E. coli* vaccination. Further detailed analysis during the period of *E. coli* vaccination demonstrated a gradual decrease in the TI_100_, as illustrated in Figure 6, where we calculated a trendline for the decrease in antimicrobial use: **y = −12.79 ln(x) + 39.64**. The limited amounts of antimicrobials that were occasionally used were directed towards *Streptococcus suis* meningitis in the second part of the post-weaning period. In contrast to previous observations [32], there was still an occasional episode of *S.-suis*-related meningitis in some weekly batches, although the clinical presence of diarrhea due to PWD was very limited to entirely absent, depending on the batch.

Mortality data were recorded in detail, keeping track of the number of dead piglets in each weekly production batch (Table 1). During P1, an average of 52 piglets died per weekly batch, resulting in a 5.7% mortality. Following stabilization of the *E. coli* PWD vaccination effect, the average number of dead piglets dropped to 16 per weekly batch, resulting in 2.0% mortality. Moreover, the fluctuation in mortality percentage that occurred during the first few weeks stabilized over the next months (Figure 3). The significant reduction in both the number of dead piglets (*p* = 0.001) and the mortality percentage (*p* = 0.004) between P1 and P2 represented an important economic value, as will be apparent in the ROI calculation table (Table 2). Since the weight of dead piglets was not available, we have no specific information on the period of and reason for death of the piglets during the post-weaning period. In contrast to previous *E. coli* vaccination studies, we could not conclude that the problem of *S. suis* meningitis in the second part of the post-weaning period disappeared in this case. Although the treatment with amoxicillin could be reduced, there was no complete resolution of the *S. suis* infection pressure, as was previously observed in other studies.

Based on the published economic ROI calculator by [32], we calculated an ROI of +EUR2.72 per vaccinated piglet, considering the cost of *E. coli* vaccination (Table 2). For this calculation, we used the collected and calculated performance parameters during P1—first 6 weeks following *E. coli* vaccination—and P2—following 16 weeks of *E. coli* vaccination. For this specific 1000-sow farm, an extra income of EUR89.746 could be generated based on improved mortality, an increased ADWG, and reduced antimicrobial use.

## 4. Materials and Methods

### 4.1. Description of the Sow Farm

The farm was a 1000-sow farm with PIC sows (PIC, Pig Improvement Company; local distributor: KI Lichtervelde, Lichtervelde, Belgium) operating in a 1-week batch management system (BMS) with 50–55 sows weaned per weekly batch, resulting in 750–850 weaned piglets every week. During lactation, the sows were fed a standard lactation diet dosed individually in 3 feedings a day and increasing from day 2 to 12 according to their individual consumption, based on a daily evaluation by the production manager. The sow farm was characterized by the following performance parameters: 17.3 live-born piglets per litter, 10% pre-weaning mortality, and 15.5 piglets weaned per litter. Sows were vaccinated during gestation for Porcine Reproductive and Respiratory Syndrome Virus (PRRSV), Influenza A Virus—Swine (IAV-S), Porcine Parvovirus (PPV), *Erysipelothrix rhusionpathiae*, *Mycoplasma hyopneumoniae*, atrophic rhinitis due to *Pasteurella multocida* DNT and *Bordetella bronchiseptica*, and neonatal *E.-coli*-associated diarrhea.

The farm was rated as an ‘attention farm’ at the level of the post-weaning period according to the Antimicrobial Consumption and Resistance in Animals (AMCRA; Brussels, Belgium) benchmark reporting tool (= above the TI_100_ threshold of 40 for post-weaned piglets). To analyze the specific approach towards antimicrobial use and the related post-weaning pathology, a farm visit including a biosecurity check was carried out together with all associated stakeholders.

Subsequently, an antimicrobial coaching trajectory was enrolled to follow up on the improvement of the reduction in antimicrobial use after implementation of the various advice.

### 4.2. Antimicrobial Coaching Trajectory

The enrolment of the antimicrobial coaching trajectory contained both a thorough analysis of antimicrobial data sheets including an interview with all stakeholders at the farm level (farm owner, farm veterinarian, and employees in different sections of the farm), and a farm visit through all sections of the farm (gilt rearing, gestation, farrowing, post-weaning facilities) to get a broad idea of the general and specific farm management techniques related to disease control and prevention and the use of antimicrobial products.

Based on the information generated, an action plan was designed with practical advice towards the improvement of general biosecurity, water management, general hygiene measures including C&D, and specific measures related to PWD. An overview of this advice is given in Table 3.

### 4.3. Post-Weaning-Diarrhea Diagnosis

The farm suffered for several years from PWD outbreaks due to ETEC in every consecutive weekly batch. This resulted in consistently high (=above the TI_100_ threshold of 40 for post-weaned piglets as defined by AMCRA) antimicrobial use during the post-weaning period related to PWD due to ETEC and subsequent meningitis due to *S. suis*. Therefore, piglets (n = 5) that remained untreated for a 6-day period with typical clinical signs of PWD, such as watery diarrhea, a thin belly, and signs of dehydration, were sampled using rectal swabs (Sterile Transport Swab Amies with Charcoal medium; Copan Italia S.p.A., Brescia, Italy). All sampled piglets were post-weaned for 3 to 5 days. The diagnostic samples were sent to the laboratory (IZSLER, Brescia, Italy) under cooled conditions for further processing.

Specimens were processed using the standard procedures for the isolation and characterization of intestinal *E. coli* [5,14]. Briefly, samples were plated on selective media and on tryptose agar medium supplemented with 5% defibrinated ovine blood and incubated aerobically overnight at 37 °C. Hemolytic activity was evaluated, and single coliform colonies were further characterized.

DNA samples were prepared from one up to five hemolytic and/or non-hemolytic *E. coli* colonies and used to perform a multiplex PCR for the detection of fimbrial and toxin genes, including those encoding for F4 (K88), F5 (K99), F6 (987P), F18, F41, LT, STa, STb, and Stx2e, but not discriminating between F4ab, F4ac, and F4ad. The methodology used for the identification of these virulence genes has been described previously [14]. All collected samples were positive for F4 in combination with STa, STb, and LT. No other virulence factors could be detected (Table 4).

### 4.4. Vaccination Protocol

Following identification and specific pathotyping of the strain involved in PWD, a practical vaccination protocol was designed to protect piglets from F4-ETEC during the ‘at risk’ period post-weaning. Therefore, an oral live avirulent *E. coli* F4/F18 vaccine (Coliprotec F4F18; Elanco Animal Health, Indianapolis, IA, USA) was diluted into cold water including 25 g of electrolytes and 0.5 g of stabilizer (AviBlue; Elanco Animal Health, Indianapolis, IN, USA) per liter of water volume. The piglets were vaccinated according to the manufacturers’ instructions at 18 days of age, approximately 7 days prior to weaning. The vaccine was administered only once on the day of vaccination.

The volume of water was determined through a volume consumption essay prior to the vaccination day. The outcome of this essay was a volume of 1.75 L per litter. In this volume, a total of 12.5 doses of live avirulent *E. coli* vaccine were diluted. From a practical point of view, a vial of 50 doses was diluted into 7 L of water with electrolytes and stabilizer. The vaccine was to be consumed by the piglets in the litter within 4 h after distribution.

### 4.5. Piglet Performance Data Capture and Experimental Design

The following data were captured from the weaned piglets: weaning weight, number of weaned piglets per batch, duration in the post-weaning facility, weight at the end of the post-weaning phase, mortality, and antimicrobial use during the post-weaning period. Based on these data, the mortality percentage and treatment incidence over 100 days in nursery (TI_100_) were calculated.

For analytical purposes, the results in the first 6 weeks post-vaccination (period 1, P1, control; vaccine stabilization period) were compared to the results in the following 16 weeks (period 2, P2, vaccine; stable vaccine period).

### 4.6. Economic Return-on-Investment Calculation

An economic return-on-investment calculation was performed based on that of [34] Vangroenweghe (2021). Therefore, the average data from the first 6 weeks of vaccination (P1; week 1–6) were compared to the following 16 weeks of vaccination (P2; week 7–22) considering the performance data, treatment and vaccination cost, opportunity cost related to mortality, and number of sows on the farm.

### 4.7. Statistical Analysis

Calculations, exploratory data analysis and quality review, and subsequent statistical analysis were all performed in JMP 16.0. All data were presented as means with their respective standard error of the mean (SEM). All means were tested for significant differences (*p* < 0.05) using a *t*-test.

## 5. Conclusions

In conclusion, implementation of several biosecurity measures in combination with the use of an oral live avirulent *E. coli* F4F18 vaccine (Coliprotec F4F18) could improve performance parameters and mortality, while reducing the number of days in nursery and the TI_100_ in a farm with an ‘attention’ status at the level of the post-weaning period according to the AMCRA benchmark reporting tool. Overall, a positive return-on-investment of +EUR2.72 could be obtained per piglet produced under these conditions. Current policies combined with the problem of antimicrobial resistance and environmental sustainability urge the need for effective strategies to control PWD due to *E. coli* in post-weaned piglets. By properly addressing the virulence factors of ETEC, novel strategies will arise with more eco-friendly approaches to control PWD that are more in line with the public health concerns regarding antimicrobial resistance (Castro et al., 2022) [36].

## Figures and Tables

**Table 1:** Comparative table of performance results from period 1 (1–6 weeks) and period 2 (7–23 weeks) of vaccination with a live avirulent *E. coli* F4F18 vaccine (Coliprotec F4F18; Elanco AH).

	Period 1	Period 2	*p*-Value
Weeks of vaccination	1–6	7–23	-
Average weaning weight (kg)	6.40 ± 0.17	7.03 ± 0.07	0.001
Number of weaned piglets per batch	851 ± 79	752 ± 29	0.069
Mortality (n)	52 ± 11	16 ± 3	0.0001
Mortality (%)	5.7 ± 0.01	2.0 ± 0.00	0.0004
Average weight at end of nursery (kg)	22.86 ± 0.49	21.97 ± 0.26	0.467
Average days in nursery (d)	44.95 ± 1.13	38.01 ± 0.74	0.002
Average daily weight gain	366 ± 5	392 ± 7	0.011
TI100 (d)	32.8 ± 9.6	20.6 ± 3	0.082
ROI per piglet		+2.72	-

**Table 2:** Return-on-investment calculation based on the production results prior to the start of the *E. coli* vaccination (Coliprotec F4F18; Elanco Animal Health, Indianapolis, IN, USA) of a 1000-sow farm with a profile of high antimicrobial use provoked by post-weaning diarrhea due *Escherichia coli* F4 and subsequent meningitis due to *Streptococcus suis*.

Coliprotec F4F18—ROI Calculator		
Input variables	Control	Coliprotec F4F18
Weaning weight (kg)	6.20	7.03
Piglet price (25 kg) (EUR/big)	EUR56.00	EUR56.00
Duration of post-weaning phase (days)	46.10	38.01
Average daily weight gain (g/day)	355.00	392.70
Feed conversion rate (kg feed/kg growth)	1.73	1.73
Feed cost post-weaning phase (EUR/ton)	EUR324.00	EUR324.00
Percentage of piglet mortality (%)	5.70	2.00
Treatment cost (EUR/day/piglet)	EUR0.05	EUR0.05
Treatment incidence (days/100 days in production)	53.10	24.10
Coliprotec F4F18 vaccine cost (EUR/dose)	EUR1.00	EUR1.00
Number of sows at the farm	1000.00	1000.00
Piglets weaned per sow per year	33.00	33.00
Return-on-investment calculation	Control	Coliprotec F4F18
Weight at end of post-weaning phase (kg)	22.57	21.96
Total feed intake (kg)	28.31	25.82
Total feed cost (EUR/piglet)	EUR9.17	EUR8.37
Treatment cost antimicrobials (EUR/piglet)	EUR2.66	EUR1.21
Supplement extra weight piglet (+25 kg) (EUR/piglet)	EUR−2.43	EUR−3.04
Opportunity cost of mortality (EUR/piglet)	EUR3.19	EUR1.12
Vaccination cost (EUR/piglet)	EUR0.00	EUR1.00
Gain per piglets (EUR/piglet)	EUR43.55	EUR46.26
Extra gain per piglet with Coliprotec F4F18 (EUR/piglet)		EUR2.72
Return-on-investment		EUR2.72
Impact Coliprotec F4F18 at farm level per year		
Total number of weaned piglets		33,000
Cost of vaccination with Coliprotec F4F18 (EUR)		EUR33,000.00
Extra farm income per year (EUR)		EUR89,746.80
Extra income per piglet weaned (EUR)		EUR2.72

**Table 3:** Overview of general and specific biosecurity measures proposed within the scope of the antimicrobial coaching trajectory to reduce the use of antimicrobials on a farm with a problematic user profile according to the AMCRA evaluation tool.

Type of Measure	Measure Description
General biosecurity	Regular cleaning and disinfection of carcass rendering location
	Cleaning and disinfection of carcass recipient after use and prior to new re-use for the next batch. Use of a disinfectant with a strong virucidal spectrum.
	Footwear management around carcass rendering location Separate boots Alternatively: use of plastic overshoes
Water management	Permanently disinfecting surface water source with a dosing pump on the waterline
	Prevent *Clostridia* growth through continuous aeration of the open-air water source combined with a peroxide disinfection in the waterline
General hygiene measures	Objective quantitative evaluation of cleaning and disinfection procedure through Rodac plates
	Needle management upon injectable treatment: regular needle renewal Piglets: renewal after every litter Weaned piglets: renewal at start of new product vial Gilts: renewal at start of new vaccine product vial
Specific PWD approach	Postpone start of group treatment until 2 d after weaning
	Stop standard use of tilmicosin premix through feed
	Stop systematic treatment with antimicrobial premix for extended periods
	In case of clinical problems, start treatment through drinking water for a short period

**Table 4:** Diagnostic laboratory results on isolation, identification, and antimicrobial resistance profile of the *Escherichia coli* strain involved in post-weaning diarrhea and the secondary clinical problem of acute mortality due to *Streptococcus suis* meningitis.

Pathogen		*Escherichia coli*
Culture morphology		Hemolytic
Adhesins/fimbriae		
	F4 (K88)	Positive
	F5 (K99)	Negative
	F6 (987P)	Negative
	F18	Negative
	F41	Negative
Toxins		
	STa	Positive
	STb	Positive
	LT	Positive
	Stx2e	Negative
Pathotype		F4-ETEC
Virotype		F4 STa STb LT

## Data Availability

Detailed data are unavailable due to privacy restrictions.

## Abbreviations

The following abbreviations are used in this manuscript:

ADWG	Average daily weight gain
AMCRA	Antimicrobial Consumption and Resistance in Animals
BMS	Batch management system
C&D	Cleaning and disinfection
*E. coli*	*Escherichia coli*
ETEC	Enterotoxigenic *Escherichia coli*
IAV-S	Influenza A Virus—Swine
P1	Period 1
P2	Period 2
PIC	Pig Improvement Company
PRRSV	Porcine Reproductive and Respiratory Syndrome Virus
PPV	Porcine Parvovirus
PWD	Post-weaning diarrhea
Q2	Quarter 2
ROI	Return on investment
*S. suis*	*Streptococcus suis*
SEM	Standard error of the mean
sIgA	Secretory immunoglobulin A
TI_100_	Treatment incidence over 100 days in production
